# Monitoring Powdery Mildew of Winter Wheat by Using Moderate Resolution Multi-Temporal Satellite Imagery

**DOI:** 10.1371/journal.pone.0093107

**Published:** 2014-04-01

**Authors:** Jingcheng Zhang, Ruiliang Pu, Lin Yuan, Jihua Wang, Wenjiang Huang, Guijun Yang

**Affiliations:** 1 Beijing Research Center for Information Technology in Agriculture, Beijing Academy of Agriculture and Forestry Sciences, Beijing, China; 2 School of Geosciences, University of South Florida, Tampa, Florida, United States of America; 3 Institute of Agriculture Remote Sensing and Information System Application, Zhejiang University, Hangzhou, China; 4 Center for Earth Observation and Digital Earth, Chinese Academy of Sciences, Beijing, China; Fondazione Edmund Mach, Research and Innovation Centre, Italy

## Abstract

Powdery mildew is one of the most serious diseases that have a significant impact on the production of winter wheat. As an effective alternative to traditional sampling methods, remote sensing can be a useful tool in disease detection. This study attempted to use multi-temporal moderate resolution satellite-based data of surface reflectances in blue (B), green (G), red (R) and near infrared (NIR) bands from HJ-CCD (CCD sensor on Huanjing satellite) to monitor disease at a regional scale. In a suburban area in Beijing, China, an extensive field campaign for disease intensity survey was conducted at key growth stages of winter wheat in 2010. Meanwhile, corresponding time series of HJ-CCD images were acquired over the study area. In this study, a number of single-stage and multi-stage spectral features, which were sensitive to powdery mildew, were selected by using an independent t-test. With the selected spectral features, four advanced methods: mahalanobis distance, maximum likelihood classifier, partial least square regression and mixture tuned matched filtering were tested and evaluated for their performances in disease mapping. The experimental results showed that all four algorithms could generate disease maps with a generally correct distribution pattern of powdery mildew at the grain filling stage (Zadoks 72). However, by comparing these disease maps with ground survey data (validation samples), all of the four algorithms also produced a variable degree of error in estimating the disease occurrence and severity. Further, we found that the integration of MTMF and PLSR algorithms could result in a significant accuracy improvement of identifying and determining the disease intensity (overall accuracy of 72% increased to 78% and *kappa* coefficient of 0.49 increased to 0.59). The experimental results also demonstrated that the multi-temporal satellite images have a great potential in crop diseases mapping at a regional scale.

## Introduction

Crop diseases significantly impact on the yield and quality of crops worldwide [1], [2]. For decades, threats from crop diseases and pests are getting serious under the context of global climate change, which pose a challenge on our food production. Acquisition of spatial distribution information of the disease over a large area can help to understand current disease infection status which is critical for loss assessment [3], [4]. Traditionally, manual inspection and field survey are still a major way to collect disease distribution information, which is not only time consuming and labor intensive, but also impossible to monitor disease occurrence and severity in a spatially continuous manner over a large area. As the most effective technology in observing ground surface physical parameters (e.g., land surface temperature) over a large area, remote sensing provides an important alternative to traditional method in obtaining disease information spatially. Its successes in disease detection and monitoring were demonstrated by a great number of studies that were conducted at leaf, canopy and field levels [5]–[10].

It is clear that the crop disease pathogens can induce changes of biophysical and biochemical parameters of plants, such as variations of several pigments, water content and canopy structure [3], [4], as well as some leaf color changes due to pustules or lesions [9], [11]. Such changes will further result in spectral responses' abnormality, such as the increase of reflectance in a red band, the reduction of reflectance in a near-infrared band, and the change of red edge optical parameters [12], [13]. Despite hyperspectral data showed good performance in detecting crop diseases [4], [8], [15], the high cost and low availability makes it difficult to be widely implemented in practice. Therefore, a great effort has been made to utilize multispectral data for mapping diseases [16], [17], [18]. On the other hand, although the high resolution imagery leads to successes in disease detection at some specific sites (e.g., experimental fields) [8], [17], [18], the high cost and limited spatial coverage of the airborne and spaceborne data restrict their implementation at a regional scale. Instead, some moderate resolution remote sensing data, such as Advanced Spaceborne Thermal Emission and Reflection Radiometer (ASTER), Landsat Thematic Mapper (TM), have a potential in detecting or mapping diseases over vast areas given their relatively low cost and wide coverage [19], [20].

While we think that spatial/spectral resolutions of remote sensing data are important, the temporal resolution is also a vital factor in monitoring plant disease/insect damage. Several attempts have been made in extracting disease/insect signals from a time series images by analyzing their temporal -trajectories of some spectral features. Liu et al. (2006) successfully monitored the oak mortality in coastal California that was caused by forest disease through multi-temporal image analysis [21]. Goodwin et al. (2008) detected the infestation of pine beetle in western Canada using a temporal sequence of Landsat data, which yielded overall classification accuracies ranging from 71% to 86% [22]. Eklundh et al. (2009) adopted MODIS time series data for mapping insect defoliation in Scots pine forest in southeast Norway. The seasonal profiles of MODIS data were found to be useful to locate insect damage patches [23]. Since the crop diseases usually develop faster than forest diseases (the development of forest diseases is usually at a seasonal and yearly step while the development of crop diseases is usually at a monthly or weekly step), a higher revisit frequency of the data (<1 week) is required for crop disease monitoring.

In practice, it is an important demand for disease control and safeguarding food security to conduct disease detection/mapping using remote sensing data at a regional scale. However, based on our knowledge, a few studies have been conducted to detect/map widely distributed crop diseases (e.g. yellow rust, powdery mildew in winter wheat [24]) at a regional scale. One possible reason to explain is lacking of desired remote sensing data sources given the frequent conflict of spatial and temporal resolutions of remote sensing data. For example, some moderate resolution data like Landsat TM and ASTER have a relatively low revisit frequency (half a month), whereas some daily revisit satellite data such as MODIS have a coarse spatial resolution. Fortunately, the advent of environment and disaster reduction small satellites (HuanJing-1A/B) that were launched by China Center for Resources Satellite Data and Applications (CRESDA) on September 6, 2008 changes such a “conflict” because the HuanJing-1A/B would provide important remote sensing data to us with a potential of mapping disease at a regional scale. As a multispectral sensor, the HuanJing CCD image (hereafter referred to as HJ-CCD) has a similar spatial resolution (30 m) and band setting to the commonly used Landsat-5 TM. Four channels of HJ-CCD cover visible and near infrared regions, which have similar band wavelengths to the four bands of TM. However, comparing with TM, a much shorter revisit period of HJ-CCD (4 days) makes it a good trade-off at both spatial and temporal resolutions, which thus allows a temporal analysis at key growth stages for crop monitoring. Therefore, we assume that the HJ-CCD should be suitable for disease monitoring at a regional scale.

As a severe disease of winter wheat, the epidemic of powdery mildew (*Blumeria graminis*) could lead to a significant yield loss and reduction in grain quality [25]–[27]. The occurrence of powdery mildew will exhibit a distinct symptom: disease pustules appear on leaves in light white to light yellow [28]. Such physiological and leaf color changes can induce corresponding spectral variation as stated by Lorenzen and Jensen (1989) [29], showing a certain increase in reflectance in visible bands whereas a reduction in near-infrared bands. This spectral response change of powdery mildew is further confirmed at leaf and canopy scales, respectively [13], [30]. As pointed by Zhang et al. (2012), the broad-band spectral features revealed a potential in estimating the disease severity of powdery mildew [13]. Therefore, the overall goal for this study is to evaluate the performance of HJ-CCD sensor in monitoring and mapping winter wheat disease (Powdery mildew (*Blumeria graminis*)) with HJ-CCD time series images and synchronized field observations (*n*  =  90 at each stage). The specific objectives for this study are to: (1) identify a set of suitable spectral features for developing models for monitoring powdery mildew at a single stage and multi-stage; (2) compare the performance of four methods: mahalanobis distance (MD), maximum likelihood classifier (MLC), partial least square regression (PLSR) and mixture tuned matched filtering (MTMF) for detecting the disease; (3) propose and evaluate a protocol for disease monitoring at a regional scale based on the multi-temporal HJ-CCD data. Test results will be analyzed and applicability and implication of the HJ-CCD data will also be discussed.

## Study Site and Image Data

### 2.1 Study site

A study site was selected from a suburban area in Beijing, China (39.78 N, 116.73 E), which covers two main food production counties, Tongzhou and Shunyi, with a total area of over 2,000 km^2^. Across October through next June, winter wheat is a major crop in the study area. The climate of the study area is characterized by high humidity and heavy rainfall, and powdery mildew disease frequently occurs in most of years. Based on historical observations made by a governmental plant protection department in the study area, the powdery mildew starts to show its symptom after the booting stage. After a rapid development, the symptom gets heavier and peaks at the grain filling stage. At the stage of plants turning yellow due to maturity, the disease characteristics gradually vanish as the milk-ripen stage starts. Therefore, the available time window for monitoring the infection of powdery mildew is approximately only one month between the start at the booting stage and the end at the filling stage. In the growing season of 2010, a forecast of powdery mildew outbreak was predicted by Beijing plant protection station at the middle of March, 2010. Thereby, a field survey experiment was arranged in this area then.

### 2.2 Image data

**Figure 1:**
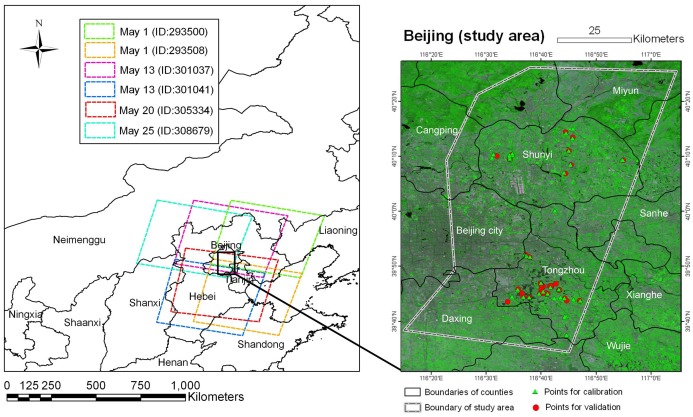
Location of survey points in the study area in Beijing, P. R. China. Left part displays the coverage of each HJ-CCD scene; right part illustrates the distribution of both training (green circle) and test survey points (red circle) in the study area. The imagery on the right side is displayed using a false color combination with R/G/B  =  Green/NIR/Red bands.

Based on the appropriate time window discussed above for the powdery mildew monitoring, only images acquired in May, 2010 were considered. After eliminating those cloud contaminate scenes, a total of 6 HJ-CCD scenes, acquired at four stages, were retained for disease monitoring. The detailed acquisition time, scene ID and illumination conditions for each scene were given in Table 1. The boundaries of all scenes were illustrated in Fig. 1. It should be noted that on May 1 and May 13, 2010, the full covered image in the study area was mosaicked by 2 simultaneously-acquired scenes.

**Table 1:** Detailed information of acquired scenes and corresponding field observations.

Stage	Date	scene ID	Path(P) & Row(R)	Acquisition time (GMT)	Illumination		FID
					Sun Zenith [°]	Sun Azimuth [°]	
Zadoks 37 (S1)	May 1	293508	P456 R68	03∶15∶10.66	26.19	328.60	Apr 30 - May 1
		293500	P456 R64	03∶14∶22.16	28.47	332.91	
Zadoks 53 (S2)	May 13	301037	P1 R64	03∶21∶41.97	25.43	330.15	May 10 - May 13
		301041	P1 R68	03∶22∶30.52	23.27	325.02	
Zadoks 68 (S3)	May 20	305334	P457 R68	03∶02∶20.90	24.30	315.99	May 19 - May 21
Zadoks 72 (S4)	May 25	308679	P4 R64	03∶28∶59.31	23.11	327.57	May 26- May 28

Acquisition time is displayed in the format of “hh:mm:ss”; FID indicates the time periods of the corresponding field observations.

## Methods

### Ethics Statement

The study area for our field survey include several parcels of normal wheat fields, which did not require any specific permission for conducting field survey. The specific location of our study area are: Longitude 116.36–117.00 and Latitude 39.56–40.44 . We confirm that our field study did not involve any endangered or protected species.

### 3.1 Image preprocessing

The preprocessing of HJ-CCD images includes a radiometric calibration, atmospheric correction and geometric correction. The calibration coefficients were provided by the CRESDA. The calibrated data were then atmospherically corrected with the algorithm provided by Liang et al. (2001), which estimated the spatial distribution of atmospheric aerosols and retrieved surface reflectance under general atmospheric and surface conditions [31]. Although all acquired HJ-CCD images were individually undertaken systematic geometric correction, a co-registration of images was implemented since pixel-based change detection requires extremely accurate spatial matching among multi-temporal images [32]. One historical Landsat ETM+ image with precisely geometric correction was used as the reference image. Each HJ-CCD image was co-registered with this reference image using over 80 ground control points. The root mean square error for each geometric corrected scene was less than 15 m.

### 3.2 Extraction of winter wheat planting area

Given the fact that the spectral divergences among different ground objects, such as farmlands, forests, water body, and impervious areas, are always greater than that between healthy and diseased areas inside a crop field, it is necessary to extract the winter wheat planting area before conducting the disease monitoring. The HJ-CCD scene acquired on May 20, 2010 (at the grain filling stage, Zadoks 68) was used for this extraction, since the winter wheat was the only crop undergoing a vigorous growth in the study area. The other crops, such as maize and soybean, were at the very beginning of their growth stages so that winter wheat areas were thereby easily separated from them. A decision tree method was adopted for this classification process. The threshold of each node was first determined by 120 field survey points with known land cover types (data not shown), and then slightly modified by a quantitative stepwise approximation method [33]. In the study area, the vegetated and non-vegetated areas were first differentiated by a threshold of 0.4 of NDVI, with pixels satisfying NDVI<0.4 classified as non-vegetation area. While in the remaining vegetated area that was consisted of farmlands, grasses and forests, the pixels satisfying NIR (near-infrared band)<0.44 were classified as grasslands. In the remaining forests and farmlands, given that the forests in the study area are distributed in mountainous area in the northwest part of Beijing, another threshold was set on elevation (using the SRTM DEM released by NASA) at 100 m. Those vegetated pixels with an elevation over 100 m were classified as forests while those pixels with elevation < 100 m as farmlands. Since the NDVI values for other crops were lower than 0.4 in May 2010, all remaining vegetated pixels in the farmlands were considered as winter wheat planting area. By comparing with the field survey points, an overall accuracy of extracting winter wheat planting area with the decision tree method reached 95%, which satisfied the accuracy requirement of subsequent analysis for disease monitoring.

### 3.3 Field inspection of powdery mildew

**Figure 2:**
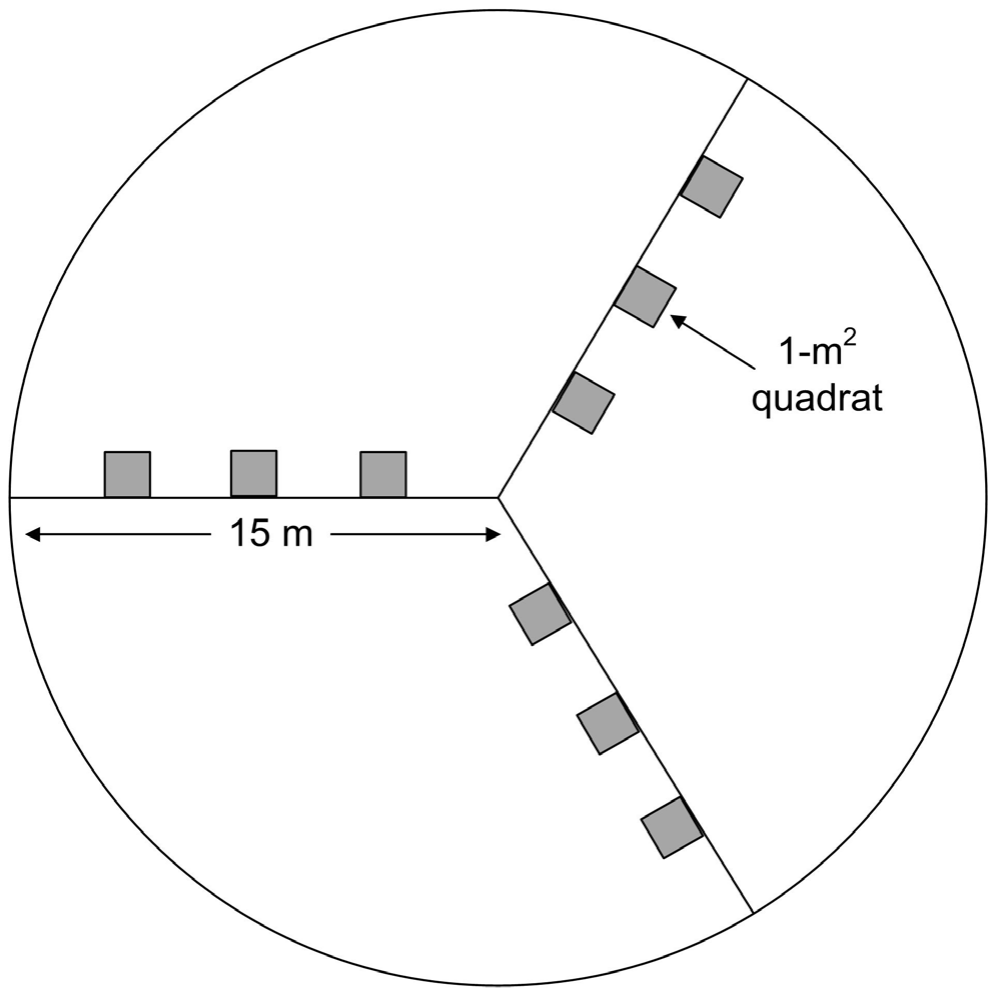
Sampling design within each plot (Modified from NAWMA field data collection scheme (Stohlgren et al., 2005)).

In the study area, a total of 90 plots were randomly selected and surveyed for disease occurrence and severity, with 54 plots for model calibration and remaining 36 plots for validation (Fig. 1). To relate the plots to corresponding image pixels, we investigated a continuously winter wheat planting region with an area of no less than 15 m in radius for each plot. The sampling design (Fig. 2) was referred to North America Weed Management Association (NAWMA) mapping standard [34].

Within each plot, a 15 m radius circle was determined as the survey region with three transects extending from the central point to the perimeter at 30° N, 150° N and 270° N (Fig. 2). Three 1-m^2^ quadrats were surveyed along each transect which made a total of 9 survey quadrats in a plot. In each quadrat, 20 individual plants were random selected for disease inspection. The disease index (*DI*) was used as an indicator of disease severity, following our predecessors [8], [35]. In disease inspection, each leaf of the selected plants were grouped into one of 10 classes of damage percentage: 0% (incidence level, x = 0), 1–10% (x = 1), 11–20% (x = 2), 21–30% (x = 3), 31–40% (x = 4), 41–50% (x = 5), 51–60% (x = 6), 61–70% (x = 7), 71–80% (x = 8),81–100% (x = 9) covered by powdery mildew by experienced investigators. Of them, 0% represents no incidence of powdery mildew whereas 100% represents the greatest incidence. The *DI* was then calculated using [35]: 
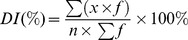
(1)


where *f* is the total number of leaves of each class of disease severity; *x* is the incidence level, and *n* is the highest incidence level. The *DI*s of 9 quadrats within a plot were then averaged to represent the disease severity of the plot. To facilitate some methods that were used for assessing disease severity using a discrete manner, we also grouped the disease plots into two disease classes by adopting a threshold of 30% of *DI*, with *DI*<30% as slightly diseased class while *DI*>30% as heavily diseased class. This criterion of *DI*  =  30% is suggested by the national plant protection department (Chinese Standard: NY/T 613-2002). Since the disease inspection is a labor intensive and time consuming work, we let 3 investigators conduct this survey simultaneously (each checking one transect). The investigators were equipped with a mobile computer with a built-in DGPS device (Trimble GeoXT), which were already installed a customized data entry sheet. This approach facilitated standardization and consistency in field data collection and also accelerated the survey process significantly. By adopting this survey method, the time for completing one round of field survey (for all plots) could be reduced from 6–8 days to 2–4 days. At key growth stages of winter wheat, a total of four rounds of field surveys were conducted from April 30, 2010 to May 28, 2010, with no more than 3 days difference from the acquisition date of the corresponding satellite images at each stage (Table 1).

### 3.4 Selection of spectral features

In addition to the four original bands of HJ-CCD, we also examined nine spectral vegetation indices (VIs) for their sensitivities to powdery mildew. They were simple ratio (SR), normalized difference vegetation index (NDVI), green normalized difference vegetation index (GNDVI), triangular vegetation index (TVI), soil adjusted vegetation index (SAVI), optimized soil adjusted vegetation index (OSAVI), modified simple ratio (MSR), non-linear vegetation index (NLI) and re-normalized difference vegetation index (RDVI) (Table 2). Some of these VIs were demonstrated to be responsible for the plant stress status, such as NDVI and TVI [36]–[39]. The other VIs, such as SR, NDVI and GNDVI were used for detecting plant diseases [36], [40]. Prior to this study, we conducted a field spectral measurement to healthy and powdery mildew infected plants at canopy level. All those selected VIs were sensitive to powdery mildew at 95% confidence level according to an independent t-test [13], [41].

**Table 2:** Definitions of spectral features that were tested in this study for monitoring powdery mildew.

Spectral features	Definition	Formular	Literatures
R_B_	Original reflectance of each band of HJ-CCD		
R_G_	Original reflectance of each band of HJ-CCD		
R_R_	Original reflectance of each band of HJ-CCD		
R_NIR_	Original reflectance of each band of HJ-CCD		
SR	Simple Ratio	R_NIR_/R_R_	Baret and Guyot, 1991
NDVI	Normalized Difference Vegetation Index	(R_NIR_−R_R_)/(R_NIR_+R_R_)	Rouse et al., 1973
GNDVI	Green Normalized Difference Vegetation Index	(R_NIR_−R_G_)/(R_NIR_+R_G_)	Gitelson et al., 1996
TVI	Triangular vegetation index	0.5[120(R_NIR_−R_G_)-200(R_R_−R_G_)]	Broge and Leblanc, 2001
SAVI	Soil adjusted vegetation index	(1+L)*(R_NIR_−R_R_)/(R_NIR_+R_R_+L); L = 0.5	Huete et al., 1988
OSAVI	Optimized soil adjusted vegetation index	(R_NIR_−R_R_)/(R_NIR_+R_R_+0.16)	Rondeaux et al., 1996
MSR	Modified Simple Ratio	(R_NIR_/R_R_−1)/(R_NIR_/R_R_+1)^0.5^	Chen, 1996; Haboudane et al., 2004
NLI	Non-Linear vegetation Index	(R_NIR_^2^_−_R_R_)/(R_NIR_^2^+R_R_)	Goel and Qi, 1994
RDVI	Re-normalized Difference Vegetation Index	(R_NIR_−R_R_)/(R_NIR_+R_R_)^0.5^	Roujean and Breon, 1995

### 3.5 Characterization of temporal change of spectral features

For those selected spectral features, their values at single stages only reflect the static condition of plants. While their temporal change information associated with the impact of disease is more important for disease detection. Therefore, in this study, apart from the spectral features selected for each single stage, we also extracted their temporal change information between two date images (acquired at two different times). Usually there are 3 commonly used forms to characterize the change information of variables: image differencing, image ratioing and image normalization [42], [43]. Given that the image normalization is able to suppress both additive and multiplicative noises, a normalization calculation was applied to measure the change magnitude of spectral features between two date images:
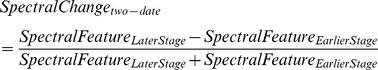
(2)


where *SpectalChange_two-date_* was a temporal change magnitude of a spectral feature between two specific stages; *SpectralFeature_EarlierStage_* and *SpectralFeature_LatterStage_* represented the spectral features at previous and later stages, respectively.

To identify spectral features that were the most sensitive to disease severity, both single-stage version and two-stage change version of spectral features were evaluated with an independent t-test. Given that the disease infections were unobvious at Zadoks 37 (S1) and Zadoks 53 (S2) stages in field, both change versions of spectral features were associated with ground surveys that were conducted at Zadoks 68 (S3) and Zadoks 72 (S4) stages, respectively. The statistical analysis was conducted with SPSS 19.0.

### 3.6 Methods used for disease monitoring

To monitor powdery mildew at a large scale, four methods were examined and compared in this study. They are mahalanobis distance (MD), maximum likelihood classifier (MLC), partial least square regression (PLSR) and mixture tuned matched filtering (MTMF). All these methods have pronouncing capabilities in classification with, and extracting information from, remote sensing data (see the references in Table 3 for detailed descriptions of the four methods). Of them, MLC is a standard parametric classifier, which assumes that the statistics for each class in each band are normally distributed. MD is a direction-sensitive distance classifier that uses statistics for each class. It is similar to the MLC but assumes all class covariances are equal and therefore is a non-parametric method [44]. The other two methods, PLSR and MTMF, were used to measure disease severity by both severity class (discrete way) and *DI* values (continuous way). Although PLSR is a modification of principal components regression (PCR), they are very different. PCR extracts factors or components to explain as much predictor sample variation as possible, while PLSR balances the two objectives of explaining as much both response variation and predictor variation as possible [45]. This method is particularly suitable for processing independent variables that present a strong correlation and redundant information among them [46], [47]. Given that most of the spectral features are constructed from reflectance of green, red and NIR bands (Table 2) in this study, a high level of correlations among the spectral features is expected. For this reason, the PLSR was chosen as one disease mapping method.

**Table 3:** Characteristics of methods used in this study for disease monitoring.

Methods	Full name	Description	Type of estimation		Literature
			by class	by DI	
MD	Mahalanobis distance	A direction-sensitive distance classifier that uses statistics for each class, which assumes all class covariances are equal.	yes		Richards, 1999
MLC	Maximum likelihood classifier	A standard parametric classifier, which assumes that the statistics for each class in each band are normally distributed and calculates the probability that a given pixel belongs to a specific class.	yes		Richards, 1999
PLSR	Patial least square regression	A statistical method that finds a linear regression model by projecting the predicted variables and the observable variables to a new space.	yes	yes	Herman, 1985
MTMF	Mixture tuned matched filtering	A method that unmixes pixels and matches pixels in the image to the endmember spectra by maximizing the target response and mini-mizing background spectral signatures.	yes	yes	Boardman et al., 1995

Unlike the PLSR, MTMF is an advanced spectral unmixing algorithm which has been successfully applied for plant species identification and crop stress mapping [18], [48], [49]. However, the effectiveness of this method in processing moderate resolution data remains unknown yet. Unlike other spectral unmixing algorithms, MTMF hypothesizes a spectral signal of a pixel as a mixture of a target and an undefined background. MTMF can be used to extract the target information from its complex background without knowing spectra of the other endmembers [48], [50]. This characteristic of MTMF is appropriate for disease detection as the disease signal is usually mixed with other possible components in the field (e.g., different cultivars and soil types). In this study, an automatic technique that combining the minimum noise fraction (MNF) with pixel purity index (PPI) was used for endmember selection (Text S1, Figure S1). Then, a spectral adjustment was implemented to modify the spectrum of selected enedmember. (Text S1, Figure S2). Outputs of MTMF include a matched filter (MF) score and an infeasibility (Inf) value. The MF score indicates the target abundance ranging from 0 to 100%, which, in our case, equals to the *DI* value. The Inf value is an estimate of the likelihood that a pixel does not contain the target signal. The higher the Inf value is, the less likely the pixel contains the target. The presentation of Inf value is to eliminate the false positives that are common to MF solutions [50]. In estimating disease severity by MTMF, a threshold of Inf should be used for removing those pseudo disease pixels. This Inf threshold can either be assigned by an expert, or be determined using a training dataset [18], [48], [49]. For PLSR and MTMF, the disease severity was measured not only by *DI*, but also by disease class, which can be referred to the same criterion as described in subsection 3.3.

### 3.7 Methods of accuracy assessment

**Figure 3:**
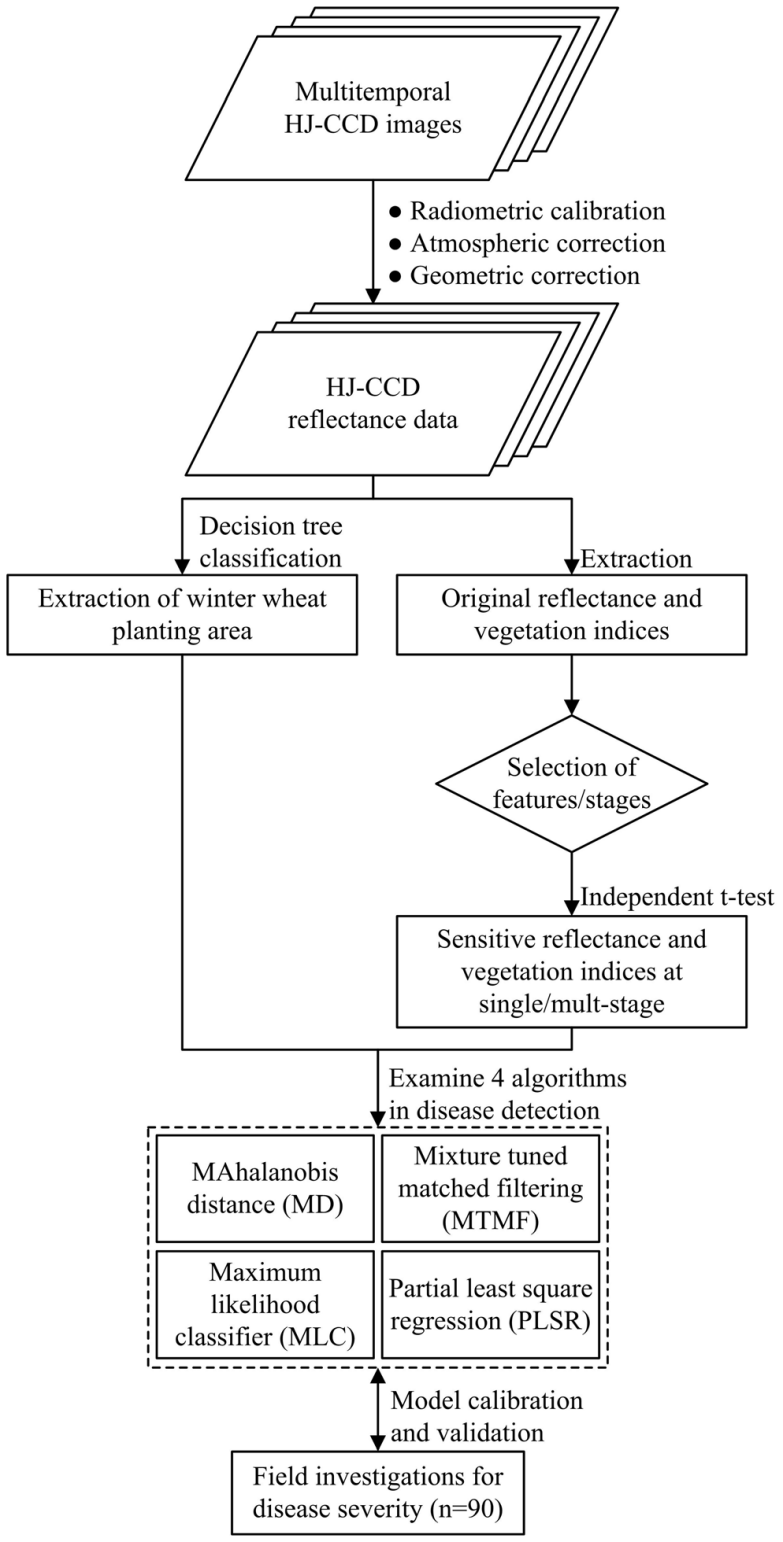
General workflow of mapping powdery mildew at a regional scale.

The performance of four methods for estimating disease severity is assessed and compared by a number of accuracy indices. They include overall accuracy, producer's accuracy, user's accuracy and *kappa* coefficient. The accuracies of results in *DI* are evaluated by two indices: the coefficient of determination (*R* ^2^), and root mean square error (RMSE). The formula of RMSE is:
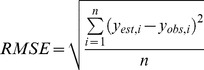
(3)


where *n* is the sample size; *y*_est_ is the estimate of *DI*; *y*_obs_ is the *DI* observation. The entire workflow of mapping powdery mildew at a regional scale was presented in Fig. 3.

## Results

### 4.1 Spectral response of powdery mildew and its development

**Figure 4:**
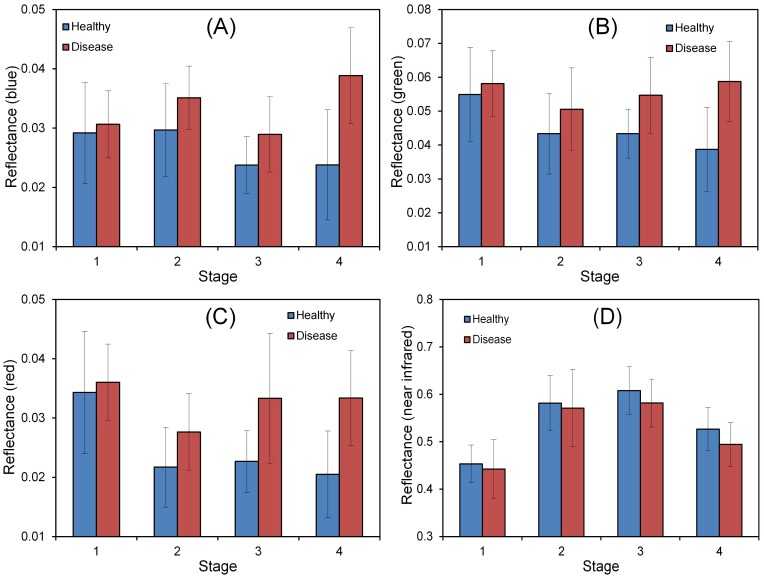
Means and standard deviations (small bars) of reflectance of each HJ-CCD band from both healthy and disease plots at different stages. A–D indicates figures of blue, green, red and near infrared channels.

**Figure 5:**
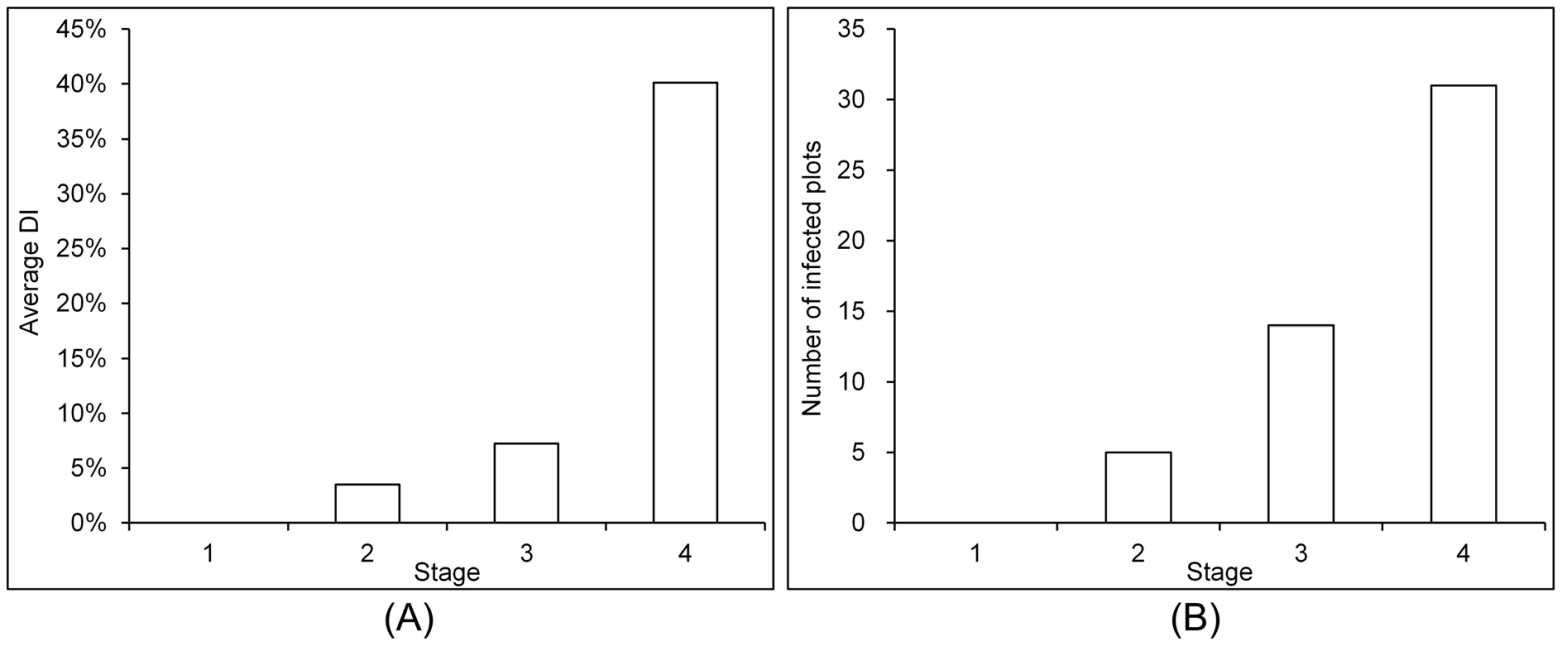
The number of infected plots and their average *DI*s at different stages (*n*  =  90).

The spectral change induced by powdery mildew infection is a basis for its remotely sensed monitoring. As shown in Fig. 4, a difference of spectral response between healthy and infected plots was compared at all growing stages by means and standard deviations of each HJ-CCD channel. Generally, all three visible channels showed higher reflectance in diseased samples than in healthy samples, whereas the NIR channel exhibited an opposite pattern. Besides, from a temporal perspective, the spectral difference between healthy and diseased samples became clearer over time. Among the 4 channels, both green and red channels showed significant spectral response difference (*p*-value<0.05 according to the independent t-test) between healthy and diseased samples since S2, whereas the blue and NIR bands failed to exhibit such a significant difference at most stages except for S4. Such temporal pattern of spectral response could reflect a disease development process, which was also evident from our field survey records (Fig. 5). All the plots without powdery mildew at S1 might only have some latent infections at this stage. The disease appeared with some visible symptoms at S2, by a few (5 out of 90) plots identified as being infected. Then, a sharp increase of both the number of disease plots and their disease severities occurred from S3 to S4, with the number of infected plots increasing from 14 to 31, and the averaged *DI* increasing from 7% to 40%.

### 4.2 Sensitivity of spectral features to powdery mildew infection

The results of independent t-tests provided a quantitative way to measure the sensitivity of each spectral feature to powdery mildew (denoted by *p*-value) at both single stage and their temporal change (Table 4). All tested spectral features showed no or weak responses to powdery mildew until S3, and thus most of spectral features were sensitive to the disease at S4. For spectral features at a single stage, some of them showed response to powdery mildew from S2, and then the response became stronger. For temporal change of spectral features, all spectral features showed some extent of response to powdery mildew within the 4 growing stages except R_NIR_ and TVI. We noted that most spectral features (11 out of 13) achieved the highest significant level such as their temporal change between Zadoks 53 and Zadoks 72 stages (S4S2) (*p*-value<0.001). Given the problem of the powdery mildew being easy to spectrally confound with other forms of stressors in the field (e.g., nutrient stresses, drought, etc.), a strict criterion with spectral features satisfying *p*-value<0.001 was retained to identify the most sensitive spectral features/stages for modeling. As a result, a total of 12 spectral features at single stages and 3 two-stage spectral features, underlined in Table 4, were selected.

**Table 4:** Responses of spectral features to powdery mildew at single and multiple stages based on the independent t-test.

Stage	R_B_	R_G_	R_R_	R_NIR_	SR	NDVI	GNDVI	TVI	SAVI	OSAVI	MSR	NLI	RDVI
Based on survey points at S3													
S1													
S2													
S3	*				*			*	*	*	*	*	*
S2/S1													
S3/S1													
Based on survey points at S4													
S1													
S2	*	*	**		**	**	*			*	**	**	
S3	**	***	***		**	***	**	*	**	**	**	***	**
S4	***	***	***	*	***	***	***	**	**	***	***	**	**
S2S1					*						*		
S3S1		*	**		**	*					**		
S4S1	**	**	**		**	**	*		*	*	**		*
S3S2						**				*	*	*	
S4S2	***	**	**		**	***	***		**	**	**	**	**
S4S3	**	**	*		*	*	**		*	*	*	*	

*indicates difference is significant at *p*-value  =  0.05; ** indicates difference is significant at *p*-value  =  0.01; *** indicates difference is significant at *p*-value  =  0.001. “S” is short form for “stage”. The multi-stage form of spectral features were calculated using a normalization process, e.g., S2S1 represents (*SpectralFeature*_S2_-*SpectralFeature*_S1_)/(*SpectralFeature*_S2_+*SpectralFeature*_S1_).

**Figure 6:**
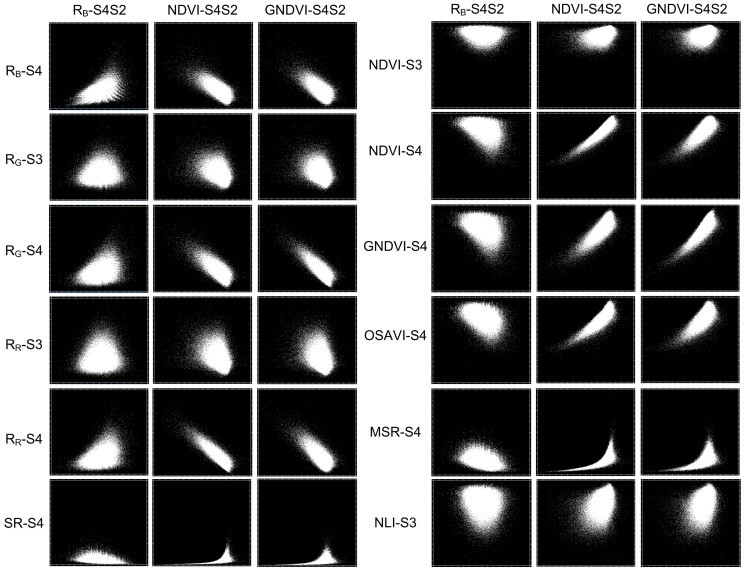
Scatter plots of all pairs of single-stage spectral features versus multi-stage spectral features. The scatter points in each plot represented all pixels within the winter wheat planting area.

In order to examine whether those identified two-stage spectral features carried additional information comparing with the single-stage spectral features, a correlation analysis was implemented for each pair of single-stage spectral features and two-stage spectral features [51]. The results showed that the coefficients of determination (*R* ^2^) between all pairs of single-stage spectral features and two-stage spectral features were lower than 0.7 (Fig. 6), which implied that the two-stage spectral features contained a certain degree of additional information. Further, we also examined whether an inclusion of those multi-stage spectral features could actually improve the mapping accuracies of powdery mildew. To do so, we compared overall accuracies of mapping powdery mildew between using models constructed with single-stage spectral features only and using both spectral features (i.e., combination of single-stage spectral features with two-stage spectral features) for all four mapping methods. The tested results demonstrated that the inclusion of the multi-stage features did improve the overall accuracies of models for all types of mapping methods, with an average overall accuracy increasing from 49% to 62%.

### 4.3 Performance of the four methods for disease monitoring

In this study, all forms of models (corresponding to the four methods) for disease monitoring were calibrated against the training data as described in subsection 3.3.

**Figure 7:**
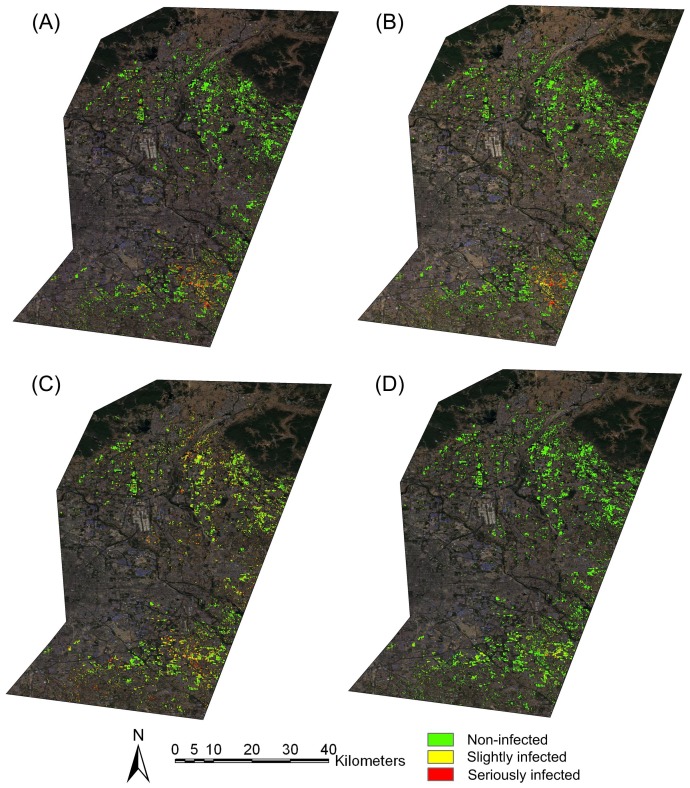
Infection map of powdery mildew produced by MD (A), MLC (B), PLSR (C) and MTMF (D).

**Figure 8:**
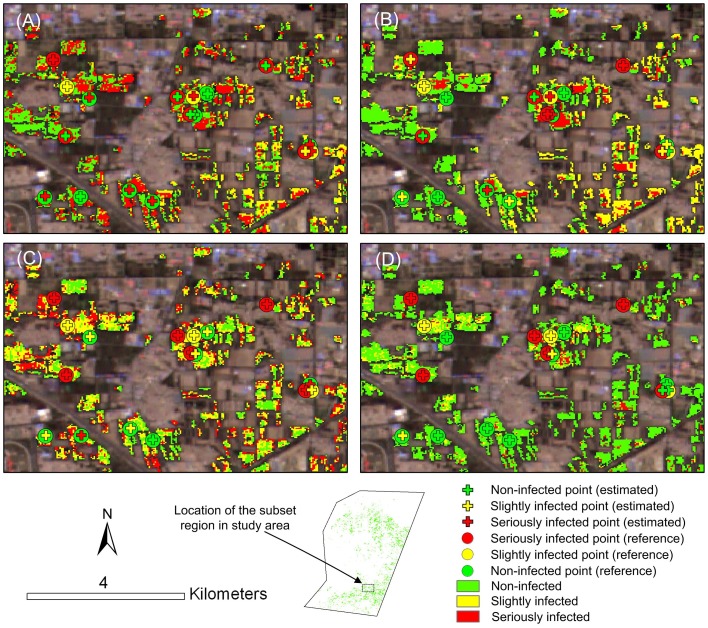
Infection map of powdery mildew in a subarea produced by MD (A), MLC (B), PLSR (C) and MTMF (D).

With all selected single-stage spectral features and two-stage spectral features, the disease maps were produced using MD, MLC, PLSR and MTMF, respectively (Fig. 7). In general, all four methods yielded similar disease distribution patterns over the study area. More infected areas and relatively higher intensity of powdery mildew were found in the southern part (Tongzhou district) than in the northern part (Shunyi district), which was in a good agreement with our field observations. Apart from the global vision of disease maps, Fig. 8 also provided a closer and more detailed vision of disease infection in a sub-region in Tongzhou county where the powdery mildew intensively occurred. Among the four methods, MD, MLC and PLSR created similar distribution patterns of disease infection areas that showed a continuous stretched pattern and occupied most of the parcels in the sub-region. However, such an infection pattern of powdery mildew was inconsistent with the pattern observed in the field. Both our field surveys and interviews with local farmers suggested that the powdery mildew exhibited a scattered pattern around the grain filling stage (S4) within the sub-region. However, such a scattered pattern was close to the infection pattern produced by using MTMF.

In discriminating between discrete disease levels, Table 5 summarizes the classification results in four confusion matrices created with the four mapping methods. From the table, it is apparent that the accuracy indices of classification varied significantly for different methods, with overall accuracy from 56% to 72% and *kappa* coefficient from 0.31 to 0.49. Among the four methods, the MTMF produced the highest overall accuracy and *kappa* coefficient. In Table 5, the user's accuracy and producer's accuracy of slightly, heavily infected and healthy classes reflected the commission error and omission error of each class. Moreover, to facilitate the visual comparison between the infection estimation and ground truth, we labeled them with cross and circle signs for each surveyed plots in Fig. 8, respectively. The infection levels were distinguished by adopting a coloring system, with healthy, slightly infected and heavily infected plots displaying in green, yellow and red colors, respectively. Referring to these symbols, for a specific plot, a correct estimation can be ensured if the colors of cross sign and circle sign were the same. For healthy class, the four methods consistently produced high user's accuracy (83–100%) and varying low producer's accuracy (36–86%), which indicated that the healthy samples tended to be misclassified as disease samples. For slightly infected class, all methods but PLSR were unable to identify the class accurately, with both user's accuracy and producer's accuracy lower than 60%. The PLSR created a producer's accuracy of 100%. Different from identifying the slightly infected samples with low accuracies, the heavily infected samples were relatively accurately identified with the four methods, with user's accuracy ranging from 43% to 100%, and producer's accuracy from 44% to 89%. In addition, the four methods exhibited different traits in discriminating normal (healthy) and infection classes. Of them, the MLC performed poorly in classifying both the healthy and diseased samples. MD yielded moderate accuracy for the healthy samples, yet low accuracies for two levels of diseased classes. The PLSR produced the highest producer's accuracy for the slightly and heavily infected classes, yet the lowest producer's accuracy for healthy class (see those yellow and red cross in green circle in Fig. 8c). Such results suggested that the PLSR had superior discriminating capability in differentiating the two infected levels, whereas performed poorly in identifying the infected samples from healthy ones. On the contrary, the MTMF produced the highest producer's accuracy for the healthy samples among the four methods, whereas performed poorly in differentiating the slightly infected and heavily infected samples.

**Table 5:** Confusion matrices and classification accuracies produced by the four methods with test samples (*n* = 36).

	Reference							
	Normal	Slight	Heavy	Sum	User's accuracy (%)	Overall accuracy (%)	Average accuracy (%)	Kappa
Classified (MD)								
Normal	16	1	3	20	80.00	61.11	52.39	0.31
Slight	0	2	2	4	50.00			
Heavy	6	2	4	12	33.33			
Sum	22	5	9	36				
Producer's accuracy (%)	72.73	40.00	44.44					
Classified (MLC)								
Normal	13	3	3	19	68.42	52.78	47.85	0.20
Slight	6	2	2	10	20.00			
Heavy	3	0	4	7	57.14			
Sum	22	5	9	36				
Producer's accuracy (%)	59.09	40.00	44.44					
Classified (PLSR)								
Normal	8	0	0	8	100.00	58.33	75.08	0.42
Slight	11	5	1	17	29.41			
Heavy	3	0	8	11	72.73			
Sum	22	5	9	36				
Producer's accuracy (%)	36.36	100.00	88.89					
Classified (MTMF)								
Normal	19	2	2	23	82.61	72.22	63.60	0.49
Slight	3	3	3	9	33.33			
Heavy	0	0	4	4	100.00			
Sum	22	5	9	36				
Producer's accuracy (%)	86.36	60.00	44.44					

Note: the fullnames of MD, MLC, PLSR and MTMF were referred in Table 4.

**Figure 9:**
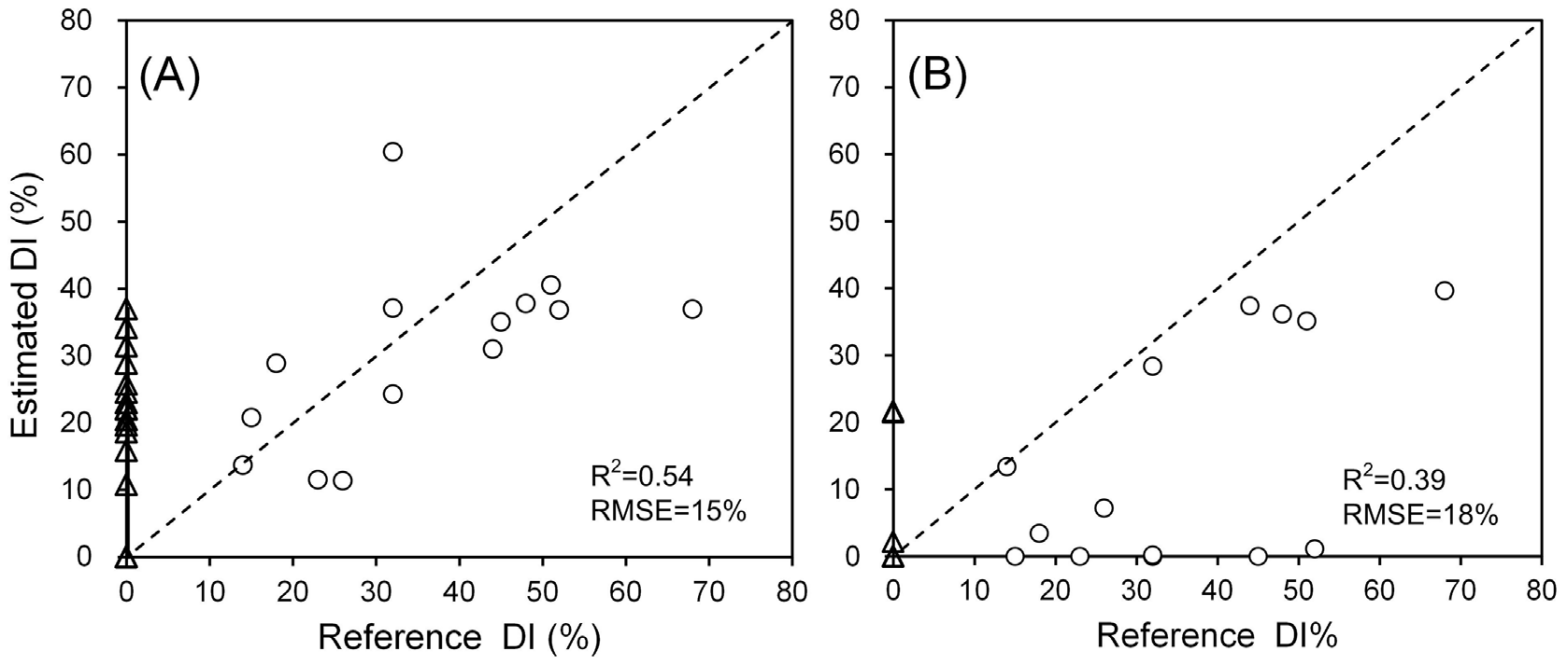
Scatter plots between surveyed *DI*s (reference) and *DI* estimates produced by PLSR (A) and MTMF (B). The circle indicates diseased samples whereas the triangle indicates normal samples. The R^2^ and RMSE are calculated based on diseased samples.

For PLSR and MTMF, apart from discrimination between discrete disease levels, both methods can provide continuous *DI* estimates (Fig. 9). Compared with the reference *DI*s, PLSR outperformed MTMF in estimating *DI*s for those disease samples (scatter plots closely distributed alone the 1∶1 line), yet also produced more errors in estimating those non-infected samples (scatter plots distributed alone the Y-axis) than MTMF. In general, both PLSR and MTMF failed to produce accurate *DI* estimates, with *R* ^2^ of 0.30 and 0.34, and RMSE of 25% and 15%, respectively.

## Discussion

### 5.1 Spectral response and temporal characteristics for powdery mildew monitoring

The pustules on leaves created by powdery mildew fungus are the most dominant symptom of powdery mildew [29]. Zhang et al. (2012) conducted a thorough spectral analysis to examine the spectral responses of powdery mildew at a leaf level [13]. Their results suggested that the powdery mildew infection could induce a significant increase of reflectance in the visible spectral region but a slight decrease of reflectance in the near infrared region. Such spectral characteristics of powdery mildew were also observed in the present study (Fig. 5). For this case, a likely explanation is the breakdown of chlorophyll pigments and cell structure, and the complication of the color of pustules due to powdery mildew [13].

In practice, there is a high possibility that the disease is spectrally confused with other types of disturbances (e.g., nitrogen shortage or drought) at individual stages. However, the temporal characteristics of disease are always unique given the different developing processes of various stressors. Therefore, theoretically the inclusion of multi-stage features may improve the mapping accuracy of the disease. A significant improvement of a multi-temporal modeling in monitoring of the disease spread compared with the single-date image classification was reported by Liu et al. (2006) [21]. Moreover, the advantages of multi-stage spectral features in disease monitoring have also been demonstrated in Goodwin et al. (2008) and Eklundh et al. (2009)'s studies [22], [52]. In this study, it was observed that the infection of powdery mildew started from a lower layer and gradually developed into an upper layer within the canopy [53], which thereby induced light visible symptoms appearing at the booting stage (S2) and the most obvious manifestation at the grain filling stage (S4). Such pronouncing temporal trait of powdery mildew development allows extracting some multi-stage spectral features outlining the process. The three multi-stage spectral features identified at S4S2: R_B_, NDVI and GNDVI, successfully captured the temporal change signals of powdery mildew, which were also demonstrated to be independent to those single-stage spectral features, thus demonstrating the usefulness of multi-stage features in disease monitoring.

### 5.2 Advantages and limitations of the four mapping methods

As shown in subsection 4.2, the four methods: MD, MLC, PLSR and MTMF exhibited different traits in mapping the intensity of powdery mildew. The MD and MLC are commonly used methods for classification of multi-spectral data. However, they performed relatively moderate in disease detection in this study. A possible reason is that the spectral difference between normal and diseased pixels is relatively smaller than that between different crops, which may not be easy to be differentiated in a low dimension feature space. An important advantage of MD and MLC is that the two methods can be driven by discrete infection classes instead of continuous *DI* estimations, which thus reduces the possible cost of conducting ground surveys.

For PLSR and MTMF, either method exhibited distinct characteristics in disease monitoring. On the one hand, the MTMF performed the best in discriminating infected and non-infected regions, with both user's accuracy and producer's accuracy of healthy class over 80% among the four methods, whereas the other three methods were characterized by high user's accuracy and low producer's accuracy of healthy class. The latter indicated a certain degree of overestimation of disease infection (Fig. 8). On the other hand, the MTMF performed poorly in differentiating slightly and heavily infected classes. The introduction of an infeasibility value for MTMF provides a means to quantify the possibility of infeasibility of the disease severity estimates given by MF results, which thus greatly prevented the influence from those pseudo infected pixels. This advantage of MTMF has also been mentioned by Franke and Menz (2007) in disease monitoring with high resolution satellite images [18]. The MTMF only took several typical infected samples into account, which did not include much spectral divergence between different infection levels. Such a defect might explain its poor performance in differentiating disease severity levels. On the contrary, the PLSR outperformed the other three methods in discriminating the slightly and heavily infected samples, but led to a significant overestimation of disease infection regions (Table 5, Fig. 8, Fig. 9). One likely reason is that the training samples used by PLSR included varied infection levels, which thus brought abundant spectral information in the model. Moreover, the PLSR used a number of principal components (instead of original spectral features) for establishing the relationship between raw data and the disease severity, which thereby eliminated the colinearity among variables, and thus enabled the accurate discrimination of different infection levels [54], [55]. Further, due to the lacking of a mechanism to eliminate the pseudo results, the PLSR model was prone to response to other unhealthy pixels rather than disease infection (e.g., nutrient stresses, drought, etc.), which thus led to a serious overestimation problem in disease monitoring.

As reported in this paper, both MTMF and PLSR methods performed better in identifying the disease occurrence and estimating the severity degree of infection respectively than the other two methods. However, given the relatively weak spectral response of disease and high level of uncertainty (e.g. planting density, confusion of several types of stresses), with multi-temporal satellite images and present methods, it is able to locate the infected areas of powdery mildew yet is still challenging to provide accurate determination of their infection levels. Considering a practical use in disease control and a requirement of monitoring the infected area at a region scale, we suggest to use MTMF method to achieve this goal.

### 5.3 An integration of MTMF and PLSR

**Figure 10:**
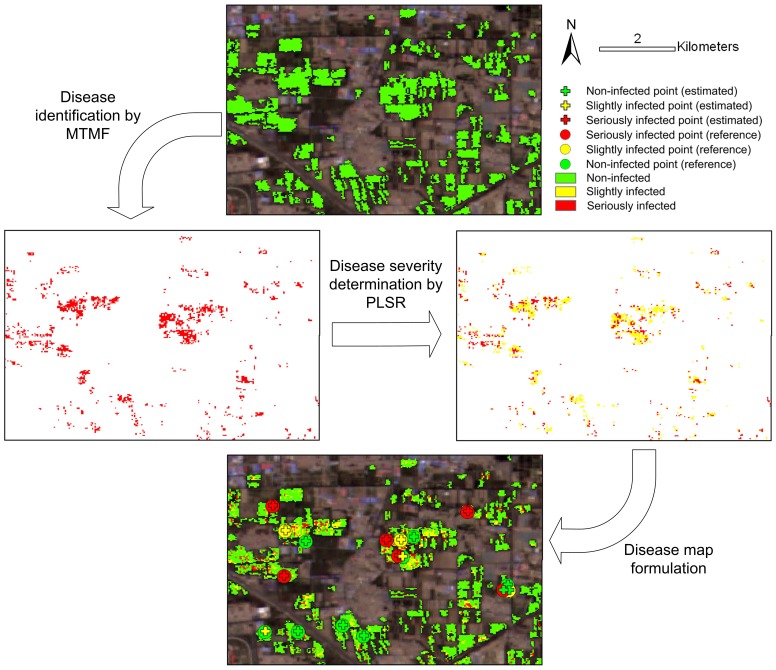
Workflow of an integration of MTMF and PLSR in mapping powdery mildew.

Based on the discussion above, since the four algorithms have had some imperfections in disease identification or severity determination, it seems that none of them alone can produce satisfactory results in this study. While we discussed the performance of the four algorithms in disease mapping (subsection 5.2), the mutually complementary traits of MTMF and PLSR in disease identification and severity determination attracted our attention and evoked us to synergize their traits for disease monitoring tasks. The processing workflow was illustrated in Fig. 10 by showing the result of each processing step for the subset region in our study area. (1) The MTMF was used to identify those infected patches by powdery mildew from healthy areas. (2) For those pixels in the infected regions, the PLSR was applied to differentiate their severity levels. The performance of the integrated model was encouraging based on its validation result of overall accuracy improved from 72% (the highest accuracy for individual algorithms as shown in Table 5) to 78% and the *kappa* coefficient improved from 0.49 to 0.59 (Table 6). In addition, when quantifying disease severity in a continuous manner, the *R* ^2^ was improved from 0.39 to 0.54 and the RMSE decreased from 18.02 to 14.80. All the aforementioned accuracy improvements in disease identification and severity determination indicated that the combination of MTMF and PLSR demonstrated the synergy of both algorithms. Therefore, it is recommended to use this integrated algorithm for disease monitoring.

**Table 6:** Confusion matrix and classification accuracies created by the integrated approach of MTMF and PLSR.

		Reference							
		Normal	Slight	Heavy	Sum	User's accuracy (%)	Overall accuracy (%)	Average accuracy (%)	Kappa
Classified	Normal	19	2	2	23	82.61	77.78	71.01	0.59
	Slight	3	3	1	7	42.86			
	Heavy	0	0	6	6	100.00			
	Sum	22	5	9	36				
	Producer's accuracy (%)	86.36	60.00	66.67					

### 5.4 Potential applications to crop diseases monitoring at a regional scale

In this study, taking PM as an example, a framework of crop disease monitoring was proposed that is able to incorporate routinely operated multi-temporal satellite images at a regional scale. It should be realized that proposed mapping strategy is actually an open system, which allows adaptations or modifications according to the spectral response and temporal characteristics of a specific disease. Different from the disease mapping at a parcel scale that aims at facilitating the precision farming management, the distribution information of disease across a certain region is vital to macro-decision making process, such as strategic planning, identifying areas requiring intensive field survey, adjusting the budget for prevention practices, allocating limited fungicides and yield forecasts [56], [57]. Besides, the information about an extent and intensity of a disease occurrence is also useful in loss assessment for agricultural insurance.

## Conclusions

With a set of time series HJ-CCD images and a corresponding intensive ground survey of disease incidence, the multi-temporal moderate resolution images could be used to map powdery mildew in a winter wheat area with an overall accuracy of 78%. In this study, both MTMF and PLSR performed better in mapping the diseased area and estimating infection severity. However, given the MTMF unique ability of identifying the infected area of powdery mildew, the MTMF method was recommended for practical use. Such mapped and estimated powdery mildew information derived from the time series satellite observations can greatly assist the assignment of further field investigations for reality check and decision making.

However, it should be noted that some limitations and challenges still remain in monitoring crop diseases with the multi-temporal moderate resolution images at a regional scale. Firstly, the technical flow for disease mapping as analyzed in this study is only suitable for some types of diseases that result in a continuous stretching landscape pattern, which thus may not be suitable for some sparsely occurred diseases (e.g., yellow rust in winter wheat). Secondly, some environmental variations, such as phonological difference, cultivation procedures and soil types, may also cause some confusions and uncertainties in disease monitoring given their similar spectral and temporal properties with disease. In our study, even using the optimal model, the total variance of over 20% was not accounted for yet. To overcome these challenges, remote sensing data combined with some ancillary data, such as meteorological data, geographic data, etc., may help reduce the uncertainty level in disease mapping process. Moreover, incorporation of physically processing models (e.g., SIR model) that describe the disease dispersal behavior and mechanism will be expected to facilitate the disease monitoring process. Therefore more studies in this field are necessary.

## Supporting Information

Figure S1 **Workflow of endmember selection.**
(TIF)

Figure S2 **The optimization of the Inf for MTMF analysis.**
(TIF)

Text S1 **Endmember selection for MTMF.**
(DOCX)

Text S2 **Spectral adjustment for “partially pure” endmember pixels.**
(DOCX)
