# Supplementary figures and images for: Monitoring Powdery Mildew of Winter Wheat by Using Moderate Resolution Multi-Temporal Satellite Imagery
Source: PLoS One. 2014 Apr 1;9(4):e93107. doi: 10.1371/journal.pone.0093107 (PMC3972229; doi:10.1371/journal.pone.0093107)

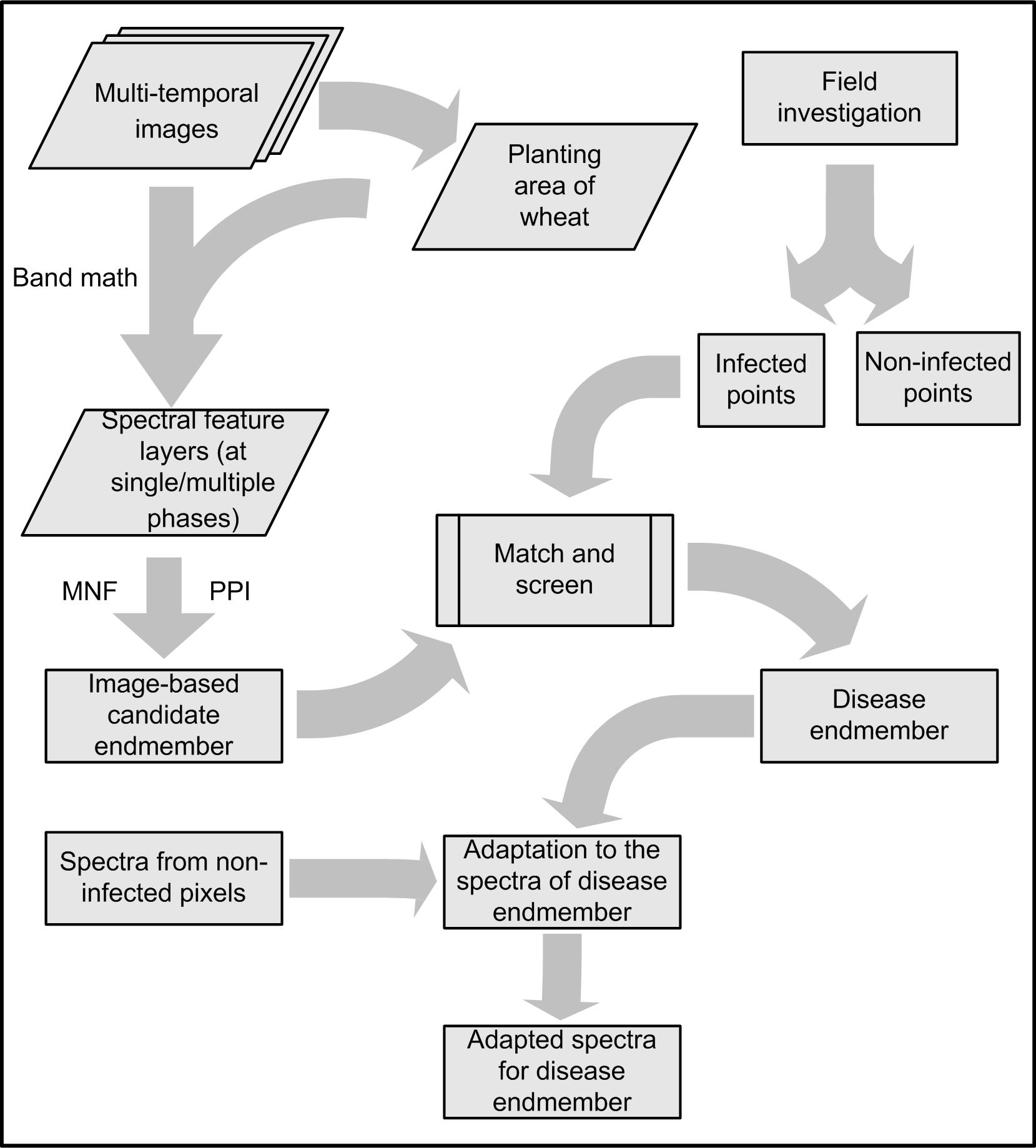

Supplement: Figure S1 — Workflow of endmember selection. (TIF) [file pone.0093107.s001.tif]

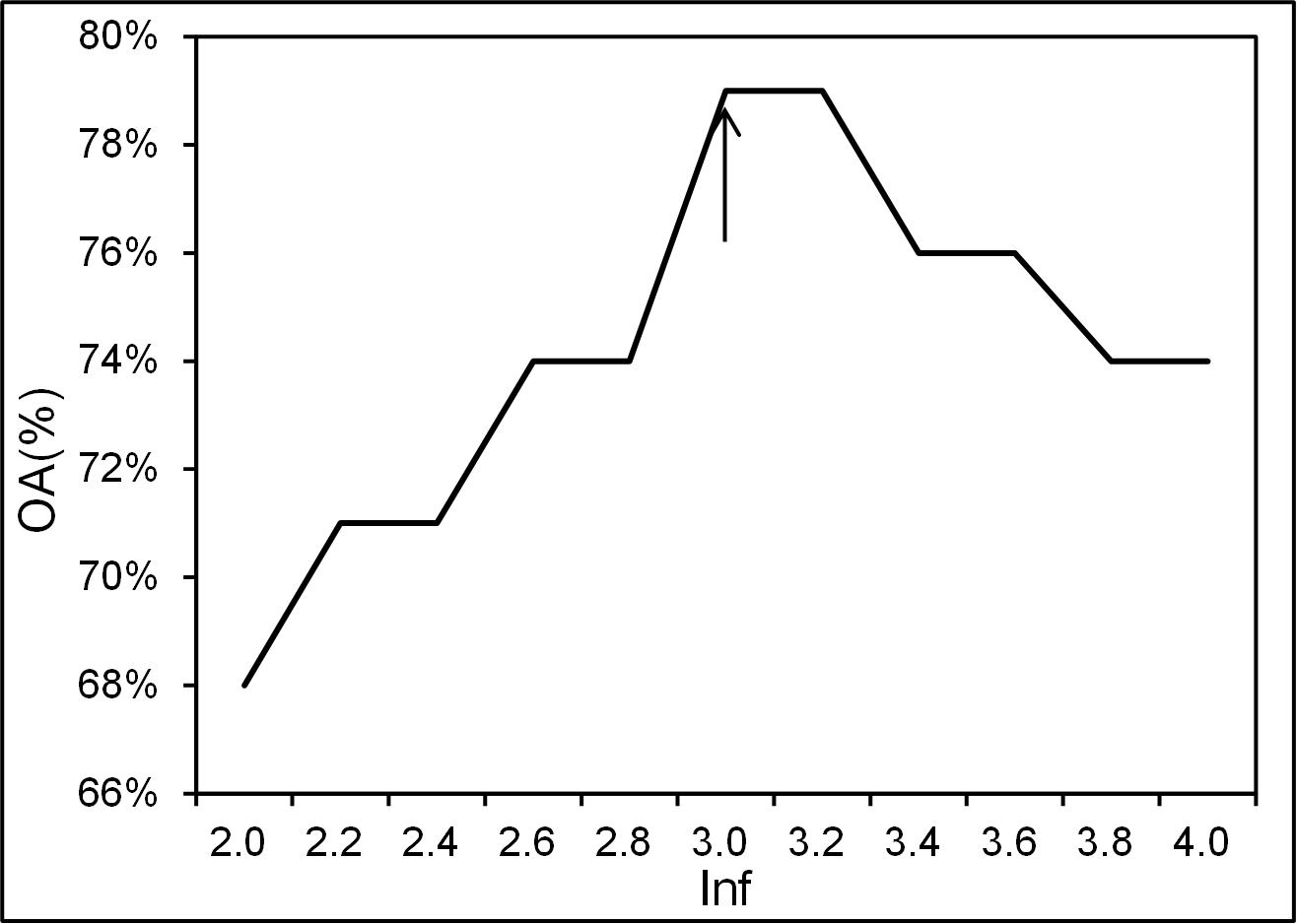

Supplement: Figure S2 — The optimization of the Inf for MTMF analysis. (TIF) [file pone.0093107.s002.tif]
