# Supplementary material for: Monitoring Powdery Mildew of Winter Wheat by Using Moderate Resolution Multi-Temporal Satellite Imagery
Source: PLoS One. 2014 Apr 1;9(4):e93107. doi: 10.1371/journal.pone.0093107 (PMC3972229; doi:10.1371/journal.pone.0093107)
Supplement: Text S1 — Endmember selection for MTMF. (DOCX) [file pone.0093107.s003.docx]

**Text S1. Endmember selection for MTMF**

In MTMF, the endmember selection is the most important step to successfully unmix a stacked image data and produce valid fractional abundances for each pixel. Improper endmember selection will lead to meaningless fraction maps which will thus result in failure of disease detection. For HJ-CCD data, the endmember (i.e., pure disease pixel) is not easily extracted given a spatial resolution of 30 m. Therefore, a rigorous procedure for endmember identification as shown in Fig. 11 was implemented in this study, which integrated both automatic and manual endmember selection methods, and proved to be efficient in extracting weak signal from images.

As shown in the left branch of the workflow in Fig. 11, the automatic technique is an image- based endmember selection process which is a combination of minimum noise fraction (MNF) with pixel purity index (PPI). The PPI function is able to calculate the spectral purity of each pixel by repeatedly projecting n-D scatter plots on a random unit vector in MNF feature space. The higher PPI score a pixel has, the less chance it is a mixed pixel. To avoid disturbances from other ground objects, the endmember selection above was only processed within the winter wheat planting area (see subsection 3.2 for its extraction) in the image. Apart from the automatic technique, a manual selection was also introduced (the right branch of the workflow in Fig. 11) to identify those pure disease endmember pixels from those image-based endmember candidates. To achieve this goal, we limited the disease endmember selection to those surveyed pixels (corresponding to surveyed plots as described in subsection 3.3). Only pixels that meet the following two conditions were selected as disease endmember pixels: (1) their surveyed *DI* values should be greater than 0.5; (2) their PPI scores should be ranked at the top 10% within the image. Based on such conditions, only five pixels were identified as disease endmember pixels in our study area. This is because these pixels contained unique powdery mildew signal and had a sufficient spectral uniqueness at the same time. All the aforementioned procedures for endmember selection were implemented in ENVI 4.3.
