# Supplementary material for: Monitoring Powdery Mildew of Winter Wheat by Using Moderate Resolution Multi-Temporal Satellite Imagery
Source: PLoS One. 2014 Apr 1;9(4):e93107. doi: 10.1371/journal.pone.0093107 (PMC3972229; doi:10.1371/journal.pone.0093107)
Supplement: Text S2 — Spectral adjustment for “partially pure” endmember pixels. (DOCX) [file pone.0093107.s004.docx]

**Text S2. Spectral adjustment for “partially pure” endmember pixels**

As for disease mapping by MTMF analysis, those identified disease endmember pixels as described in Appendix 1 should not be directly used as targets as they were still partially pure disease endmember pixels. The perfect target pixels (i.e., disease pixels) should be covered by 100% severe diseased plants, which in other words should have a DI value equals to 1.0. However, it is almost impossible to find such pixels in the field. Instead in this study, those identified as disease endmember pixels only had DI values ranging from 0.52 to 0.72. To solve this problem, a spectral adjustment method was cited and implemented to convert those partially pure disease endmember pixels to theoretically pure disease endmember pixels. To conduct this conversion, we assume that the spectral features are linearly related with DI value. Then, the spectral feature of pure disease endmember can be calculated based on the spectral feature of partially pure disease endmember, healthy endmember and the DI of the partially pure disease pixel (plot):

$SpectralFeature_{partial}=SpectralFeature_{pure}\times DI+SpectralFeature_{healthy}\times\left(1-DI\right)$ (4)

which can be re-written as:

$SpectralFeature_{pure}=\frac{SpectralFeature_{partial}-SpectralFeature_{healthy}+SpectralFeature_{healthy}\times DI}{DI}$ (5)

The SpectralFeaturehealthy is an averaged value of the corresponding spectral feature of healthy endmember pixels, which were also selected from the surveyed pixels that were free of disease and also had the PPI scores ranked at the top 10% of the image. After such an adjustment, those theoretically pure disease endmember pixels were finally used as targets in the MTMF analysis.

For determination of Infeasibility (Inf, detailed see subsection 3.5) for MTMF, a quantitative stepwise approximation method was used to identify an ideal Inf threshold by varying Inf thresholds ranging from 2.0 to 4.0 (because most pixels’ Inf value fall in 2.0-4.0) with a step of 0.2. Through this analysis, an optimal Inf threshold of 3.0 was determined when the highest overall accuracy (79%) reached.
